# The necessary future of chiropractic education: a North American perspective

**DOI:** 10.1186/1746-1340-13-10

**Published:** 2005-07-07

**Authors:** Lawrence H Wyatt, Stephen M Perle, Donald R Murphy, Thomas E Hyde

**Affiliations:** 1 Division of Clinical Sciences, Texas Chiropractic College, 5912 Spencer Highway, Pasadena, TX, 77505 USA; 2 Division of Clinical Sciences, University of Bridgeport College of Chiropractic, 126 Park Avenue, Bridgeport, CT, 06604 USA; 3 Rhode Island Spine Center, 329 Wickenden Street Providence, RI 02903 USA; 4 Department of Community Health, Brown University School of Medicine, Box G-A, Providence, RI 02912 USA; 5 2240 NE 202 St, Miami, FL 33180 USA

## Abstract

The chiropractic educational system in North America is currently in a state of flux. The attempted conversion of some chiropractic schools into "universities" and the want of university affiliation for chiropractic schools suggests that we are searching for a better alternative to the present system. In the early 20^th ^century, the Flexner Report helped transform modern medical education into a discipline that relies on scientific and clinical knowledge. Some have wondered if it is time for a Flexner-type report regarding the education of doctors of chiropractic. This article outlines the current challenges within the chiropractic educational system and proposes positive changes for that system.

## Background

The chiropractic educational system in North America is currently in a state of flux. Proposed programs such as the Florida State University School of Chiropractic and the conversion of some chiropractic schools into "universities," suggests that we are searching for a better alternative to the *status quo*.

Medical education in the early twentieth century underwent substantial change. Published in 1910, The Carnegie Foundation Bulletin 4, "Medical Education in the United States and Canada" is widely acknowledged as the study that resulted in the reformation and reconstruction of the entire medical educational system. The report renounced the plethora of private and proprietary medical schools of that era, and established scientific medicine and clinical teaching within a university system as the gold standard for teaching medicine. This report, submitted by Abraham Flexner, is more commonly known as the Flexner Report. Although criticism of the report has resulted in some alterations to the original post-Flexner system of medical education, the reliance on scientific and clinical knowledge remains the base of the professional education of medical doctors.

Just before the publication of the Flexner Report, the Council on Medical Education had conducted a similar survey of all medical schools in North America. They essentially graded the existing medical schools at the time as A, B, or C according to a number of criteria including educational requirements, curriculum, and resources.

To a large extent, Flexner's study of medical education at the turn of the century was an exercise in inescapable conclusions. It was less a pragmatic study aimed at unearthing problems within medical education than it was a fact-finding task dedicated to verifying the prevailing view of medical school academia and its insufficient base in science. Essentially, the answers to questions regarding the inadequacy of education were already well-known. Flexner simply graded the schools based on this knowledge.

Perhaps the greatest mystique of the Flexner Report is how successfully the recommendations were followed. This report changed the educational system of an entire profession and many suggest that no other single study has been as visibly successful in accomplishing what the Flexner Report did. One reason for the enormous impact of the report was the huge financial resources that were allotted to those schools that followed the recommendations of the report. Monies from various philanthropists funded the growing expenses of the limited number of medical programs that met the standards.

In the current period of inconsistency and controversy in chiropractic education, it is not surprising that we hear a call for a Flexner-style report in our educational system. Such a critical and comprehensive examination of all the existing programs, one would hope, would weed out ineffective practices and programs in our schools and result in a clear set of recommendations for the future of science-based chiropractic education. This idea is not new, having been proposed in a similar fashion by John J. Nugent, DC in the early to mid 20^th ^century.

This paper explores, through descriptive literature analysis and the author's experiences within the North American chiropractic educational system, the current status of chiropractic education in North America. Special consideration is given to the essential role of chiropractic governing bodies, the essential history of chiropractic education, including an overview of educational standards, curricula, externship and postgraduate training programs, along with evidence-based health care and the development of chiropractic researchers. Suggested changes to the North American chiropractic educational system are explored including higher admission standards, the need for a chiropractic college admissions test and admission interviews, creation of a research culture in chiropractic schools, support by chiropractic educational and governmental regulatory agencies and the qualifications of chiropractic school administrative staff. In addition, we explore the need for strong postgraduate residency-based educational programs to enhance the exposure of students to a larger volume and variety of patients.

## Discussion

### The Current Status of US Chiropractic Education

A profession is defined by a specialized body of knowledge requiring advanced training and by the dedication of its practitioners to the public good over their own enrichment. In exchange, professionals are granted considerable autonomy in setting standards and in the conduct of their work. Any professional level educational system must adopt the tenets of the academy: scientific thinking, rigor and critical analysis. Faculty in the academy have the dual duties of being teachers and scholars. Scholarship is the development of new knowledge, synthesis of the current state of knowledge, applications of that knowledge and teaching that incorporates that knowledge [1].

The commitment of chiropractic schools boards of regents/trustees and administrations to this paradigm of the academy and thus, promoting faculty scholarly activity, is vital to the effectiveness of the institutions. Without the support of the regents/trustees and administrators, the faculty is placed in a situation where it is difficult, at best, to provide the modern education necessary in an ever-changing evidence-based health care environment.

Early chiropractic education included classes in some basic and clinical sciences along with philosophy of chiropractic. Performance of chiropractic students on basic science boards suffered as evidenced by a 23% pass rate for chiropractic students on these board exams. Medical students during this same period (1927–1953) had an 86% pass rate [2].

In North America C.O. Watkins, D.C., Joseph Janse, D.C., A.E. Homewood D.C. and others, sought to upgrade the profession by asking serious questions about the effects of spinal manipulation on human health and they recognized that a research base was vitally important to our future. Dr. Watkins was one of the pioneers in the National Chiropractic Association's efforts to raise educational standards in the 1930s and 1940s. He demonstrated sincere concern over the image of the profession and he surmised that the development of a scientific base for chiropractic care was critical to our acceptance. Dr. Janse was appointed dean of The National College of Chiropractic. He was also a key figure in the founding of chiropractic's three most prominent US regulatory bodies: the National Chiropractic Association's Council on Chiropractic Education (forerunner of the CCE), the National Board of Chiropractic Examiners (NBCE) and the Federation of Chiropractic Licensing Boards (FCLB). He was noteworthy for his research on spinal biomechanics, sacroiliac joint function, and the treatment of posture and gait abnormalities.

In the USA in 1974, the Council on Chiropractic Education (CCE) was federally recognized as the agency for accreditation of programs and institutions offering the doctor of chiropractic degree. The current CCE "seeks to insure the quality of chiropractic education in the United States by means of accreditation, educational improvement and public information. CCE develops accreditation criteria to assess how effectively programs or institutions plan, implement and evaluate their mission and goals, program objectives, inputs, resources and outcomes of their chiropractic programs" [3]. Those schools that failed to meet the CCE standards no longer operated. All chiropractic colleges had achieved accreditation by 1995 and all also now hold accreditation with regional accrediting agencies for their baccalaureate programs and some for master's degree programs as well.

While the standards for chiropractic education have advanced over the years, there remains much work to be done. Doxey and Phillips, in their paper on entrance requirements to the various professional health care disciplines demonstrated that chiropractic colleges have the least stringent matriculation requirements [4]. Currently, only one chiropractic college requires a baccalaureate degree as an admission requirement. Seven states currently require a baccalaureate degree before granting a chiropractic license and seven have it under consideration, but few of these require that the degree was acquired before entering chiropractic school [5]. There is currently no required chiropractic college admission test.

Undergraduate training in chiropractic school consists of approximately 4,200 clock hours of didactic and practical education, with the last year spent treating patients, in some cases while still attending classes. There is only one chiropractic college in the U.S. that follows the academic standard of two semesters per year. Trimesters or quarter systems of education within chiropractic were used in an effort to reduce the time spent in school.

In general, the first four to five academic terms are spent studying basic sciences while also learning the basics of spinal examination and treatment. Terms five through eight are spent in clinical classes such a diagnostic imaging, clinical neurology, physical examination, geriatrics, pediatrics, case management and the like. In addition, it is during these terms that students refine their diagnostic and treatment skills for the management of joint diseases, primarily of the spine.

Currently, internship (more correctly externship) in the chiropractic profession is a one-year undergraduate endeavor, while it is a three to five year post-graduate program in medical and osteopathic training, including residency training. Some foreign chiropractic programs, such as Switzerland, mandate a one-year externship for recently graduated chiropractors before they are allowed to practice on their own. In addition, clerkships are routine in medical training, while they are not in chiropractic schools, although some chiropractic schools have had clerkship programs for students in lower terms. A number of chiropractic schools now offer hospital rotations to chiropractic externs. In these programs, externs spend a number of weeks working with MDs and DOs in specialty areas such as radiology, orthopedics, sports medicine, family practice, rheumatology and neurosurgery. Our cumulative observations suggest that the obvious contrast in numbers of patient encounters in a chiropractic externship, when compared to a medical/osteopathic internship, are sadly disconcerting from the perspective of the volume and variety of patient exposures. Post-graduate residencies are available to chiropractors, but residency-based training is not currently a requirement, or even commonplace, the exception being diagnostic radiology training leading to diplomate status.

Chiropractic externs are currently required to complete 250 joint manipulations, 20 complete history and physical examinations, 20 radiology studies and 15 complete patient workups, from admission to discharge, during their last year in chiropractic school (externship) while treating outpatients. The CCE is mandating that these numbers increase incrementally over the next 6 years to a total of 35.

Often these outpatients seen by chiropractic externs are friends and family members, some of whom are even paid by interns to attend the clinics for care. Nyiendo and Haldeman give credence to this finding in a study in 1986 where they concluded that "patients [in a chiropractic college teaching clinic] are not truly representative of patients seen by chiropractors in the field; they are relatively young, with mild complaints." The study concludes by suggesting that these students' clinical training may not reach the level that is necessary to manage patient problems in active practice after graduation [6]. Nyiendo confirmed these findings in 1990 [7]. Further investigation suggests that these patient types are consistent amongst chiropractic school clinics [8].

Instruction in evidence-based medicine (EBM) in American chiropractic schools also appears to be lacking. A search of the current literature finds only one study dedicated to teaching evidence-based health care in a chiropractic school [9]. One study on the use of EBM was performed in a community of chiropractors. The authors demonstrated substantial success in reducing radiography rates in patients with acute low back pain after educating the chiropractors about the current evidence for this intervention. The authors admit that the methods were quasi-experimental [10].

The Foundation for Chiropractic Education and Research (FCER) has helped to foster a research mentality and has developed a program that supports the training and development of chiropractic researchers. A number of chiropractic schools have received federal research grants but the number of researchers and grants appears to still be very small.

### Suggested Changes to the Chiropractic Educational System

In our opinion CCE needs to make the admissions standards more stringent, including the requirement for a baccalaureate degree prior to admission and the use of a chiropractic college admissions test. Some believe that increasing the difficulty of entry into chiropractic college would cause a dramatic decrease in enrollment. While we are certain that there would be a "period of readjustment," every increase in standards to date has eventually resulted in a return to previous enrollment levels as the potential students now strive to reach an attainable, but obviously elevated, bar for admission.

Mandatory interviews of applicants for chiropractic college admission would do much to help ascertain the background, breadth of knowledge, social skills and communication skills of applicants. Of course, this process will only work if it is used as a screening tool, where only the best applicants are accepted into the programs and those deserving rejection for valid reasons are actually rejected.

The curricula of colleges need to be evidence-based, which probably will mean that certain unsupported beliefs and theories of the past will, of necessity, be abandoned. In particular, this means relegating much of the dogmatic, so-called, chiropractic philosophy, which was developed as nothing more than a legal tactic to prevent incarceration of chiropractors in the early twentieth century for practicing medicine without a license, to a class on the history of the profession.

Students who perform poorly in chiropractic colleges should not be allowed to pass through the system essentially unabated, as happens currently in some institutions. We feel it unacceptable for chiropractic students to make any academic progress with grades of 'D' or 'F' on their transcripts. Such students should be given one chance at remediation and if unsatisfactory grades are achieved in the same class again or in other classes, these students should be expelled from the college. Some schools are moving to an 'A, B, C, F' grading scale. While it may seem harsh, a learned and distinguished health care profession has little room for, nor should it tolerate, academic underachievement.

While each college needs to have an active research department, all members of the faculty must accept their responsibilities as scholars. Our professional educational programs can no longer remain isolated from the academic community. Joining established research universities will help change the culture of the chiropractic professorate to one which values scholarship and models the joy of learning and discovery for their students. A "publish or perish" mentality for faculty, we suggest, would be a healthy and refreshing change.

Administrative and board support for educational objectives is crucial for any substantive improvement in the training of new chiropractors. Often, chiropractic schools have hired administrators who have little or no formal training in education, providing more political, budgetary and marketing expertise than academic experience. High-level administrators with training in education, along with administrators who have political, budgetary and marketing experience, should become the norm in chiropractic programs.

In addition to striving for university affiliation, our institutions must also endeavor to become less and less tuition-dependent. The current tuition-dependent system carries the burden of much of what is wrong with our current system. It fosters academic underachievement, admission of probably under-qualified, if not unqualified, students and under-funded research and faculty development programs.

Probably of most critical importance in making positive change in our current educational programs is the establishment of mandatory post-graduate internships and residencies with hospital and interdisciplinary training. Exposure to a large volume and variety of patients is critical to our students training if the profession is to take a place at the center of our mainstream health care system. Interns and residents must be routinely exposed to patients with conditions that represent the full spectrum of potential diagnoses that are considered by chiropractors. This first hand, on-the-job experience by new chiropractors, not just via didactics or textbook exposure, is paramount to the best clinical experience available. Certainly hospital rounds would be a great advantage in this respect. Rigorous post-graduate residencies, such as is the case currently for radiology, need to be developed to train our brightest new doctors to be leaders

## Summary

The chiropractic profession must improve itself through higher educational standards, intellectual honesty and inter-disciplinary co-operation and research rather than continue to rely on patient testimonials and political friendships. We can only obtain cultural authority when we have brought our educational programs up to the level that the public expects of an expert, learned profession. Positive changes, including a chiropractic college admissions test, elevated chiropractic school entrance requirements and mandatory post-graduate residency-based training are suggested.

## Authors' contributions

LHW wrote the initial draft of this manuscript. All authors, thereafter, made substantial contributions to recomposing the manuscript as well as appraising it critically for its chief intellectual content. Each author has given approval of the final manuscript.

## References

[B1] Boyer EL (1990). Scholarship reconsidered: Priorities of the Professoriate.

[B2] Moore JS (1993). Chiropractic in America.

[B3] Council on Chiropractic Education. http://www.cce-usa.org.

[B4] Doxey TT, Phillips RB (1997). Comparison of entrance requirements for health care professions. J Manipulative Physiol Ther.

[B5] Federation of Chiropractic Licensing Boards. http://www.fclb.org.

[B6] Nyiendo JA, Haldeman S (1986). A critical study of the student interns' practice activities in a chiropractic college teaching clinic. J Manipulative Physiol Ther.

[B7] Nyiendo J (1990). A comparison of low back pain profiles of chiropractic teaching clinic patients with patients attending private clinicians. J Manipulative Physiol Ther.

[B8] Nyiendo J, Phillips RB, Meeker WC, Konsler G, Jansen R, Menon M (1989). A comparison of patients and patient complaints at six chiropractic college teaching clinics. J Manipulative Physiol Ther.

[B9] Fernandez CE, Delaney PM (2004). Applying evidence-based health care to musculoskeletal patients as an educational strategy for chiropractic interns (a one-group pretest-posttest study). J Manipulative Physiol Ther.

[B10] Ammendolia C, Hogg-Johnson S, Pennick V, Glazier R, Bombardier C (2004). Implementing evidence-based guidelines for radiography in acute low back pain: a pilot study in a chiropractic community. J Manipulative Physiol Ther.

